# Baicalein Induces Mitochondrial Autophagy to Prevent Parkinson's Disease in Rats *via* miR-30b and the SIRT1/AMPK/mTOR Pathway

**DOI:** 10.3389/fneur.2021.646817

**Published:** 2022-02-14

**Authors:** Min Chen, Li Peng, Ping Gong, Xiaoli Zheng, Tao Sun, Xiaoqiao Zhang, Jiangtao Huo

**Affiliations:** ^1^Department of Geriatrics, Taihe Hospital, Hubei University of Medicine, Hubei, China; ^2^Department of Surgery, Traditional Chinese Medicine Hospital, Guizhou, China

**Keywords:** Parkinson's disease, baicalein, mitochondrial autophagy, striatum, miR-30b-5p, AMPK/mTOR pathway, SIRT1

## Abstract

Parkinson's disease (PD) is a prevailing neurodegenerative disorder. Baicalein has neuroprotective effects on PD animals, but its mechanism is not clarified. We explored baicalein effects on PD rats. PD rat models were established by injecting 6-hydroxydopamine into the striatum of substantia nigra on the left side of the rat brain and treated with baicalein. Dopamine (DA) content, neuronal apoptosis, neuronal injury, neuronal mitochondria, and autophagy were assessed. Baicalein-treated PD rats were treated with autophagy inhibitor 3-methyladenine to identify the role of autophagy in PD. PD rats were injected with AgomiR-30b-5p or sh-SIRT1 plasmids and treated with baicalein. PD rats elicited decreased neurological score and DA secretion of the striatum, increased neuronal apoptosis, and injury, and reduced number of mitochondria and autophagy, whereas baicalein alleviated neuronal injury and partly recovered mitochondrial dysfunction, 3-methyladenine inhibited the protection of baicalein. miR-30b-5p was elevated and SIRT1 was diminished in PD rats and inhibited by baicalein. miR-30b-5p targeted SIRT1. miR-30b-5p overexpression or SIRT1 silencing annulled the protection of baicalein. The phosphorylation level of AMPK in the substantia nigra of PD rats was decreased and mTOR was increased, whereas baicalein annulled these trends. Briefly, baicalein activated mitochondrial autophagy *via* miR-30b-5p and the SIRT1/AMPK/mTOR pathway, thus protecting PD rats.

## Introduction

Parkinson's disease (PD) is a prevailing progressive neurodegenerative disorder featured by Lewy body formation and dopaminergic neuronal death in the substantia nigra (1–3). It is a prevalent neurodegenerative disease that influences 1 to 2 out of 1,000 of the global population, especially in the aged population (4, 5). PD is induced by dopaminergic neuron degeneration or pathophysiologic loss in the substantial nigra of midbrain and neuronal Lewy bodies development, with aging, family history, pesticide exposure, and environmental chemicals as the recognized risk factors (6). The complicacy of PD has brought great clinical challenges, such as the difficulties in definitive early diagnosis, advanced-stage management, and treatment obstacles which delay the progression (7). The progression of PD is affected by neuroinflammation (8). Energy production through mitophagy and the mitochondrial electron transport chain are the 2 principal processes affected in the development of PD (9). Mitochondria are the core energy center for cell movement, participating in physiologic functions and maintaining metabolism balance (10). Mitochondria have diverse properties, including producing biosynthetic intermediates and promoting stress responses of cells, such as apoptosis and autophagy (11). Mitochondrial autophagy is a primary cellular activity and its insufficiency can lead to many aging-related diseases, especially PD (10, 12, 13). However, currently, drugs for PD management only relieve symptoms and show various adverse effects, without the ability to hinder neurodegeneration (14). Further investigation is warranted to identify PD pathogenesis in terms of mitochondrial autophagy.

There are many researchers who focus on discovering useful neuroprotective agents, especially natural neuroprotective agents (15). Baicalein is a major active flavonoid discovered from Scutellaria baicalensis, which possesses various pharmacological functions, including relieving inflammation, oxidative stress, and apoptosis, and as a result, baicalein has therapeutic potentials for PD (16). Baicalein activates mitochondrial autophagy of cardiomyocytes (17).

Baicalein is a natural compound that impacts the levels of microRNAs (miRNAs) (18), which are regarded as novel regulators for cell metabolism, proliferation, and apoptosis (19). Importantly, many miRNAs are involved in PD diagnosis, pathogenesis, and treatment (20). For example, hsa-miR-30b-5p plays imperative roles in neurodegenerative diseases, especially in PD (21). miR-30b-5p also shows high expression in treated PD patients (22). However, the function of miR-30b-5p in mitochondrial autophagy in PD is largely unknown. Mitochondrial autophagy is activated in cells through the activation of SIRT1 (23). The Bhlhe40/SIRT1 axis regulates mitochondrial autophagy in the neural stem cells (24). SIRT1 mediates the AMPK/mTOR pathway to induce mitochondrial autophagy (25).

However, whether baicalein modulates miR-30b-5p and mitochondrial autophagy *via* the SIRT1/AMPK/mTOR axis, thus protecting against PD, is less studied. This study set out to study the specific action of baicalein on PD and to provide new options for PD treatment.

## Materials and Methods

### Ethics Statement

All procedures were ratified by the ethics committee of Taihe Hospital and strictly implemented under the Guide for the management and use of laboratory animals. All efforts were taken to minimize the number and pain of included animals.

### Laboratory Animals

Totally 72 specific pathogen-free grade adult male Sprague-Dawley rats (7–10 weeks, 230–260 g) were acquired from the experimental animal center of Guangzhou Sun Yat-sen University [SCXK (Guangdong) 2016-0029, Guangzhou, China]. The rats were raised in 12-h light/dark cycles at 23 ± 1°C with 50–65% humidity, with 4 rats in 1 cage and freely available water and food. After 1 week of adaptive feeding, the rats showed no abnormality in neurobehavioral tests, and then, the experiments were carried out.

### PD Modeling Methods and Animal Treatment

As previously mentioned (26), a PD rat model was established by the injection of neurotoxin 6-hydroxydopamine (6-OHDA) into rat brain left striatum using a rat brain stereotaxic apparatus. Briefly, rats were anesthetized with 1% 50 mg/kg pentobarbital, the hair over the head was cut off, and the head was fixed on a stereotaxic apparatus. Following disinfection with iodine, rat scalp, subcutaneous tissues, and periosteum were cut. The located skull was accurately drilled using a dental drill without damaging the dura mater. The 6-OHDA solution (8 mg, 4 mL, Sigma-Aldrich, St. Louis, MO, USA) dissolved in 0.02% ascorbic acid saline was then injected into the left medial forebrain bundle (4.3 mm posterior to the bregma, 4.3 mm lateral to the midline, and 8.2 mm beneath the dural surface) at 0.5 mL/min using a 10-mL Hamilton microsyringe that was withdrawn slowly after staying in the brain for 5 min to avoid backflow along the injection track.

After 3 weeks of the modeling, the 96 rats were averagely assigned to 8 groups: (1) the sham group (all procedures were performed, and 6-OHDA was replaced with a same amount of normal saline); (2) PD group (PD rats were injected intraperitoneally with 10% DMSO diluted with the same amount of normal saline as the drug treatment group); (3) PD + Bai group (PD rats were injected intraperitoneally with 100 mg/kg baicalein, and the dosage of baicalein in other treatment groups remained the same); (4) PD + Bai + 3-MA group (PD rats were intervened by injected intraperitoneally with baicalein and 100 mg/kg autophagy inhibitor 3-methyladenine); (5) PD + Bai + AgomiR-NC group (before PD modeling, rats were injected with Agomir-NC through lateral ventricle, and after 3 weeks of modeling, rats were injected intraperitoneally with baicalein); (6) PD + Bai + AgomiR-30b-5p group (before PD modeling, rats were injected with AgomiR-30b-5p through lateral ventricle, and after 3 weeks of modeling, rats were injected intraperitoneally with baicalein); (7) PD + Bai + sh-NC group (rats were injected with sh-NC through lateral ventricle based on the PD + Bai group), and (8) PD + Bai + sh- SIRT1 group (rats were injected with sh-SIRT1 through lateral ventricle based on the PD + Bai group). Baicalein was injected once a day for 7 days. The dosage and method of baicalein were slightly adjusted according to the previous studies (27, 28). AgomiR-30b-5p and its control, sh-SIRT1, and its empty plasmid were acquired from Genscript (Nanjing, Jiangsu, China). After the treatment, all rats were scored for neurobehaviors and then euthanized by intraperitoneally injecting with 1% pentobarbital (800 mg/kg). The intact brain tissues of rats were collected, and the brain striatum and substantia nigra were separated for subsequent detection experiments. The striatum of 6 rats from each test group was used for DA detection, the striatum of the other 6 rats was made into frozen sections for TUNEL and fluoro Jade B (FJB) staining, a small part of substantia nigra of 6 rats was harvested for transmission electron microscope (TEM) observation and the activity detection of mitochondrial complex I and adenosine triphosphate (ATP), and the rest was used for tissue homogenate for western blot (WB) and reverse transcription quantitative polymerase chain reaction (RT-qPCR).

### Neurobehavioral Score

After treatment, all rats were subjected to neurobehavioral scoring. All behavioral tests were conducted in the daytime, and acclimatization training was conducted 3 days before to prevent the rats from being anxious and panic that affected the results.

In the rotarod test, rats were put on a roller rotating at 4 rollings/second, and the speed was gradually expedited at 0.3 rollings/second and the timing began. As soon as the rats fell from the roller, the timing stopped and the duration from the acceleration to the falling was recorded in seconds. The test was conducted every 1 min for 5 times to take the mean value.

In the grid test, rats were placed on a horizontal metal mesh (12 × 12 cm, 1 cm apart), which was turned 180 degrees. The timing began after all 4 paws gripped the metal mesh and stopped when 4 paws fell from the grid. It was recorded as 180 s when the hanging time surpassed 180 s. The test was conducted every 1 min for 5 times to take the mean value.

### High-Performance Liquid Chromatography-Electrical Chemistry (HPLC-EC)

The isolated brain striatum was put into an Eppendorf tube and supplemented with 0.1 m/L perchloric acid solution to fully break the tissue cells. After centrifugation for 20 min at 4°C and 14,000 g, appropriate amount of tissue supernatant was diluted and examined on the machine. The concentration of DA was calculated using the standard curve made by DA standard with the unit as pmole/mg tissue. The data were normalized to the sham group.

### TUNEL Staining and FJB Staining

The substantia nigra of rat brains was fixed for 24 h with 4% paraformaldehyde, dehydrated using 15 and 30% sucrose, and embedded with OCT freezing medium. Next, the tissues were sliced into frozen sections at 15 μm, stained following the instructions of TUNEL kits (Abcam, Cambridge, MA, USA), incubated for 5 min with 4',6-diamidino-2-phenylindole, and observed under a Leica fluorescence microscope (×200). Meanwhile, injured neurons were assessed by FJB staining (Histo-chem., Jefferson, AR, USA). The sections were first incubated with 0.06% KMnO_4_ and then with 0.001% FJB and photographed using Hamamatsu Nanozoomer 2.0 HT (Olympus, Tokyo, Japan). Six sections were collected from each tissue for observation, and 5 visual fields were arbitrarily collected from each section for counting. The results were expressed as the percentage (%) of TUNEL staining or FJB staining-positive cells to the total number of cells, and the average value was regarded as the final result.

### Ultrastructure of Neurons in Substantia Nigra Observation

The rats were euthanized by intraperitoneal injection of 1% pentobarbital (800 mg/kg), and 1 mm^3^ of substantia nigra tissues was quickly removed and fixed with 2.5% glutaraldehyde at 4°C for 2 h. After fully washing with PBS, the samples were fixed with 1% Russian acid. After fully washing with PBS, the samples were dehydrated with 30, 50, 70, 90, and 100% ethanol for 15 min, and propylene oxide was added to replace the ethanol for 30 min. Then, the samples were embedded with Epon 812 epoxy resin (Ted Pella, CA, USA), placed in the 1:1 mixture of Epon 812 epoxy resin (formula: Epon 812: DDSA: NMA: DMP30 = 27.5:4:20:0.75) and propylene oxide for 2 h, and in the 2:1 mixture for 1 h, then soaked in pure epoxy resin Epon 812 for 2 h, and placed into a 60°C oven for 12 h. The embedded tissues were prepared into 50- to 70-nm-thick ultrathin sections using an ultrathin slicer (Leica EM UC7, Leica, Germany). With 100-mesh copper mesh as the carrier mesh, the sections were stained with uranyl acetate and lead citrate and observed under a TEM (HITACHI H-7650, HITACHI, Japan) to observe the ultrastructural changes in neuronal mitochondria in substantia nigra of rats.

### Activity Detection of Mitochondrial Complex I and ATP

Rat brain substantia nigra homogenate was collected. Mitochondria were extracted and purified using the tissue mitochondrial extraction kit (130-097-340, Miltenyi Biotec, Germany). The protein concentration of mitochondrial was detected using the bicinchoninic acid (BCA) protein concentration detection kit (P0012, Beyotime, Shanghai, China). The activities of complex I (A089-1-1, Nanjing Jiancheng Bioengineering Institute, Nanjing, Jiangsu, China)_and ATP (A095-2-1, Nanjing Jiancheng Bioengineering Institute) in mitochondrial were detected using kits according to the instructions. The unit of complex I was nmol NADH min^−1*^mg^−1^ protein and the unit of ATP was nmol/mg protein.

### WB

The substantia nigra of rat brains was homogenized at 1:10 (w/v) in the homogenate buffer supplemented with a protease inhibitor cocktail. After centrifugation (12,000 g, 10 min), the protein lysate was determined using the bicinchoninic acid (Bio-Rad, Hercules, CA, USA). Protein isolation was performed by 10% SDS-PAGE (Beyotime, Shanghai, China) and moved to polyvinylidene fluoride membranes (Millipore, Billerica, MA, USA). Following 2-h blockade with PBS buffer + 5% skim milk powder, membranes were probed overnight with primary antibodies at 4°C, and with the secondary antibody IgG (1:5000, ab205718, Abcam, UK) for 2 h. Enhanced chemiluminescence kits were adopted to visualize the protein bands, and ImageJ software was employed for quantitative analyses. The primary antibodies were LC3 (1:2000, ab192890, Abcam), P62 (1:1000, ab109012), SIRT1 (1:1000, ab189494), AMPK (1:1000, ab32047), phospho-AMPK (p-AMPK, 1:500, ab131357), mTOR (1:10000, ab134903), phospho-mTOR (p-mTOR, 1:1000, ab137133), and GAPDH (1:10000, ab181603). GAPDH was employed as an internal reference.

### RT-QPCR

TRIzol reagent (Invitrogen, Carlsbad, CA, USA) was utilized to extract total RNA from substantia nigra homogenate. cDNA template was synthesized by extracting RNA using the TaqMan miRNA RT kit (ABI, CA, USA). qPCR amplification was conducted with SYBR Premix Ex Taq. GAPDH or U6 was applied as an internal parameter, and the 2^−ΔΔCt^ method was employed to calculate the levels of miR-30b-5p and SIRT1. The data were normalized by the sham group. Primer sequences are listed in Table 1.

**Table 1:** Primer sequences for RT-qPCR.

**Gene**	**Forward 5'-3'**	**Reverse 5'-3'**
*miR-30b-5p*	CACCAGCCATGTAAACATCC	ATGCTTGTTCTCGTCTCTGT
*U6*	ATTGGAACGATACAGAGAAG	GGAACGCTTCACGAATTTG
*SIRT1*	ACCGATGGACTCCTCACTAA	ATCTGCCACAGCGTCATATC
*GAPDH*	CAAGCAACTGTCCCTGAG	TAGACAGAAGGTGGCACA

### Bioinformatics Analysis and Dual-Luciferase Assay

The binding sites of miR-30b-5p and SIRT1 were searched on the bioinformatics online website TargetScanHuman7.2 (http://www.targetscan.org/vert_72/). The wild-type (SIRT1-WT) and mutant-type (SIRT1-MUT) plasmids were constructed by cloning the binding and mutated sequences into the pGL3 luciferase vector. The 293T cells were seeded into 6-well plates for 24 h at 2 × 10^5^ cells/well. After that, constructed vectors were comanipulated with mimic NC or miR-30b-5p mimic (miRNA-mimic 20 nM) (Genechem, Shanghai, China) into 293T cells using Lipofectamine 2000 (11668-019, Invitrogen). The luciferase activity was determined using the Dual-Lucy Assay kit after 24 h.

### Statistical Analysis

SPSS21.0 (IBM Corp. Armonk, NY, USA) was employed for data statistical analysis. Experimental data were depicted as mean ± standard deviation. Independent *t*-test was applied for pairwise comparisons, and one-way ANOVA was applied for multigroup comparisons. Tukey's test was adopted for the *post hoc* test. *p-V*alue was attained from a bilateral test, where *p* < 0.05 indicated statistical significance.

## Results

### Baicalein Had a Protective Function in PD Rat Models

Baicalein has neuroprotective functions in PD animal and cell models (29–31). However, its mechanism was not fully clarified. To study the effect, a PD rat model was established by injecting neurotoxin 6-OHDA into the striatum of rat brains, followed by baicalein treatment. The neurological scores of rats were first evaluated. In comparison with sham-operated rats, PD rats presented with severe neurological decline and defects in motor and sensory coordination, whereas baicalein partially relieved the nerve injury (Supplementary Figure S1A). DA content in the striatum of rat brains was assessed by HPLC-EC, which found decreased DA content in the striatum of PD rats, whereas baicalein restored DA content to some extent (Supplementary Figure S1B). The rat brain neuronal apoptosis was further detected by TUNEL staining, which showed facilitated neuronal apoptosis in PD rats, whereas baicalein hindered the apoptosis (Supplementary Figure S1C). FJB staining indicated that neuronal injury of PD rats was aggravated, but baicalein alleviated the injury (Supplementary Figure S1D). Overall, baicalein was protective in PD rats.

### Baicalein Improved Mitochondrial Disorder and Activated Mitochondrial Autophagy

**Figure 1:**
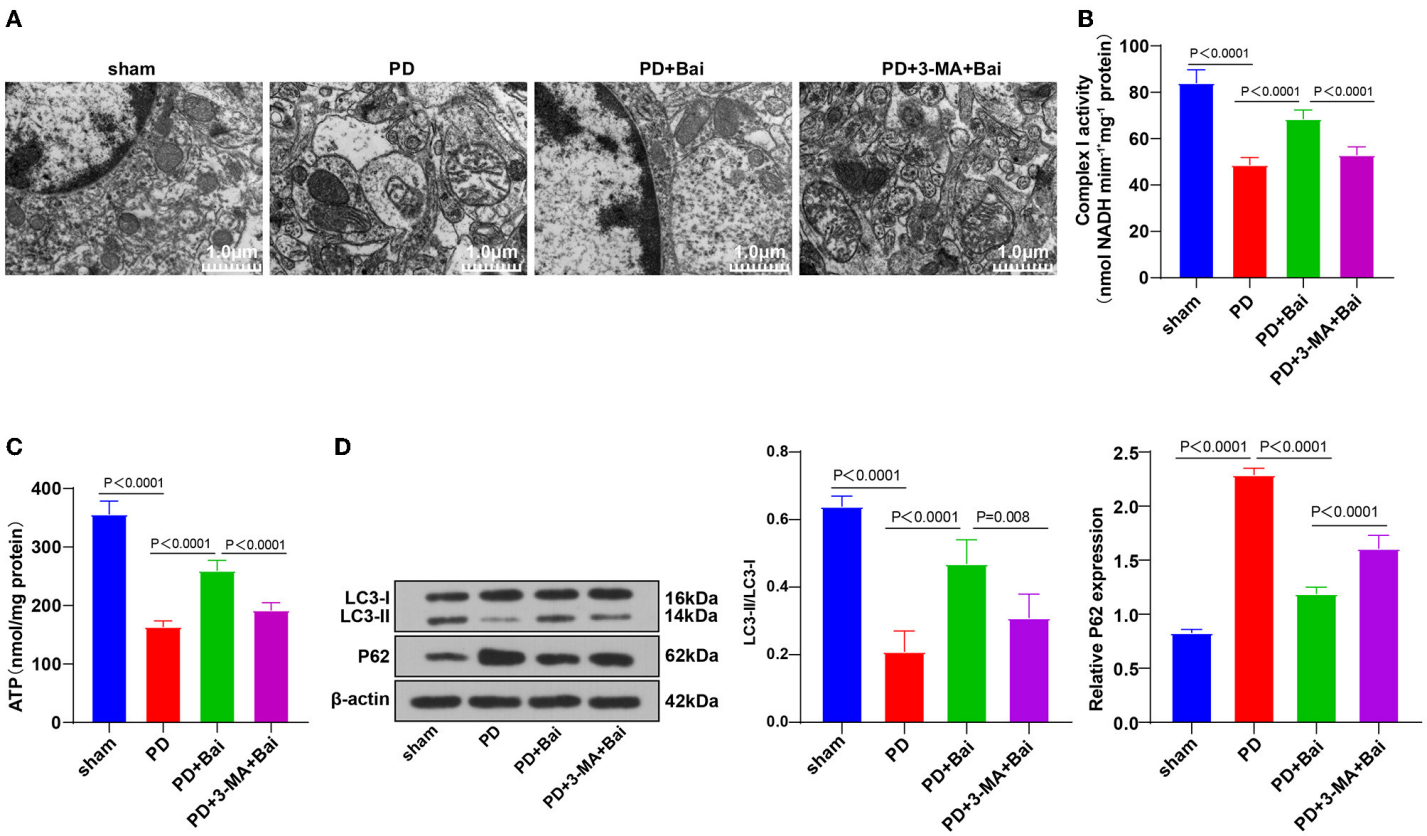
Baicalein played a protective role by promoting mitochondrial autophagy. PD rats were jointly intervened by baicalein and autophagy inhibitor 3-MA. **(A)** The ultrastructure of mitochondria was observed using TEM; **(B,C)** the activities of mitochondrial complex I and ATP were detected using kits; **(D)** the levels of autophagy marker proteins LC3-II/I and p62 were detected by WB. *N* = 6. The data in the figure are all measurement data and expressed as mean ± standard deviation; one-way ANOVA was used for variance analysis; Tukey's multiple comparisons test was used for the *post hoc* test.

The pathogenesis of PD is closely associated with mitochondrial dysfunction (9, 32). The mitochondrial ultrastructure of neurons in substantia nigra of rats was observed using a TEM. Mitochondria number in the sham group was large, dispersed, and distributed, and the morphology was regular and complete, with clear internal and external membrane structure and mitochondrial ridge structure, whereas the PD group displayed swelling, unclear double-layer membrane structure, and disordered and blurred mitochondrial ridge. After baicalein treatment, the number of mitochondria in PD rats was partially restored, and the morphology was closer to the sham group (Figure 1A). The activities of complex I and ATP (key markers of mitochondrial function) were further examined, which were repressed in the PD group, but partially restored by baicalein (Figures 1B,C). These suggested that baicalein improved mitochondrial dysfunction of neurons in substantia nigra of PD rats.

Mitochondrial disorder in PD is closely related to mitochondrial autophagy imbalance (33). The levels of mitochondrial autophagy-related marker proteins LC3-II/I and p62 were detected by WB. Relative to the sham group, LC3-II/I was suppressed and p62 level was stimulated in the PD group, indicating the limited mitochondrial autophagy, whereas baicalein upregulated the LC3-II/I and lowered p62 level, indicating that baicalein partially restored mitochondrial autophagy (Figure 1D). Furthermore, PD rats were treated with autophagy inhibitor 3-MA and baicalein. The function of baicalein in protecting PD rats was suppressed after 3-MA treatment (Figures 1A–D). Briefly, the protecting function of baicalein on PD rats might be concerned with mitochondrial autophagy.

### Baicalein Repressed miR-30b-5p and miR-30b-5p Overexpression Inhibited the Promotive Function of Baicalein on Mitochondrial Autophagy

**Figure 2:**
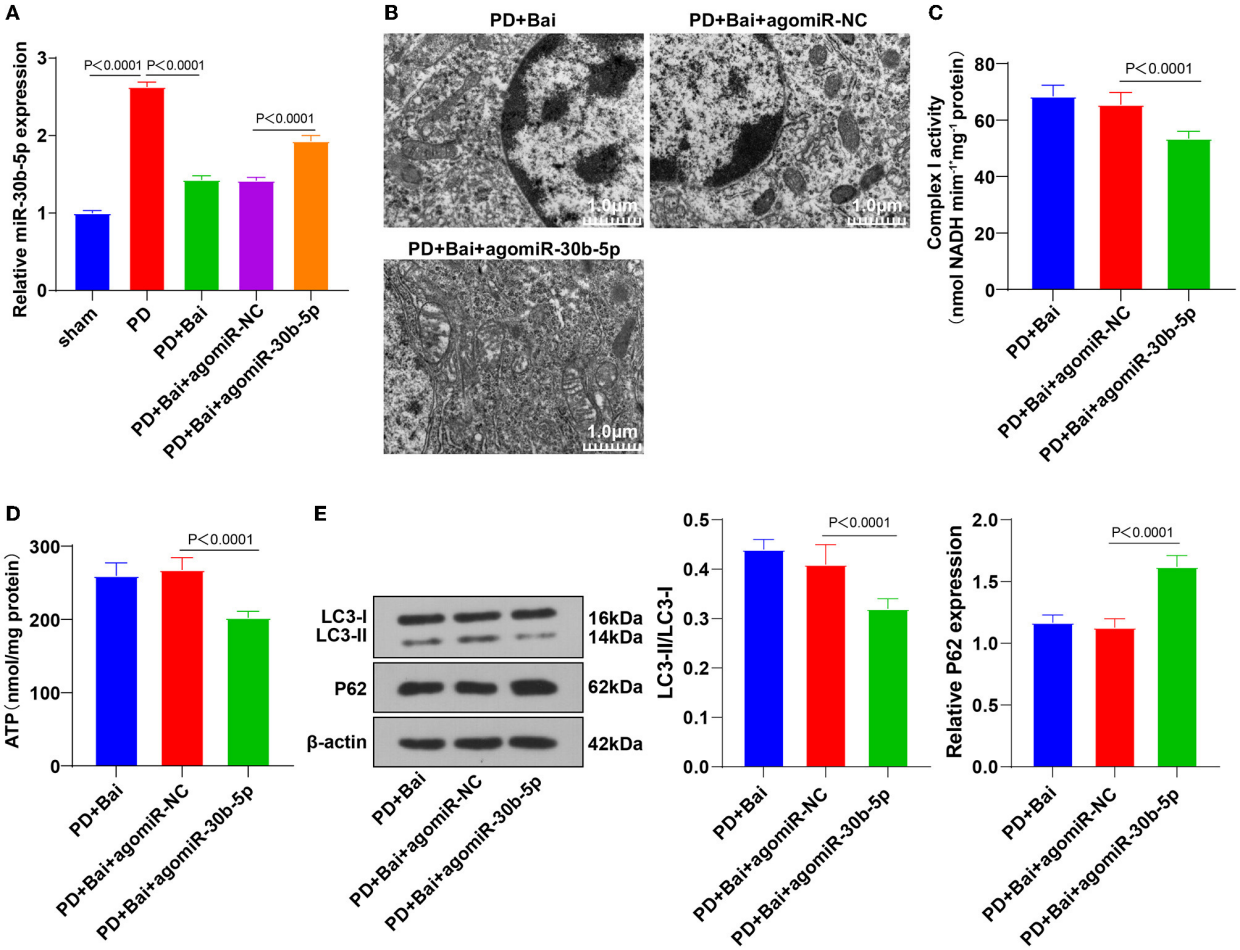
Upregulation of miR-30b-5p reversed the promotive effect of baicalein on mitochondrial autophagy. The agomiR-30b-5p was transfected into PD rats. **(A)** The expression of miR-30b-5p was detected by RT-qPCR; **(B)** the ultrastructure of mitochondria was observed using a TEM; **(C,D)** the activities of mitochondrial complex I and ATP were detected using kits; **(E)** the levels of autophagy marker proteins LC3-II/I and p62 were detected by WB. *N* = 6. The data in the figure are all measurement data and expressed as mean ± standard deviation; one-way ANOVA was used for variance analysis among multigroups; Tukey's multiple comparisons test was used for the *post hoc* test.

The abnormal expression patterns of the miR-30 family in PD patients and its regulation in autophagy have been documented (21, 22, 34). We speculated that miR-30b-5p was a potential therapeutic target of PD. miR-30b-5p expression in neurons in substantia nigra was assessed by RT-qPCR, which revealed elevated miR-30b-5p in the PD group, whereas baicalein suppressed its upregulation (Figure 2A), indicating the potential of miR-30b-5p as a target of baicalein. Thereafter, PD rats were injected with AgomiR-30b-5p to overexpress miR-30b-5p and treated with baicalein. RT-qPCR demonstrated that miR-30b-5p was overexpressed successfully. The ultrastructure of mitochondria was observed using a TEM, and the activities of mitochondrial complex I and ATP were detected. Overexpression of miR-30b-5p averted the property of baicalein on improving mitochondrial function (Figures 2B–D). The levels of autophagy-related marker proteins LC3-II/I and p62 were further detected. Overexpression of miR-30b-5p inhibited baicalein-mediated activation of neuronal autophagy in PD rats (Figure 2E).

### miR-30b-5p Targeted SIRT1

**Figure 3:**
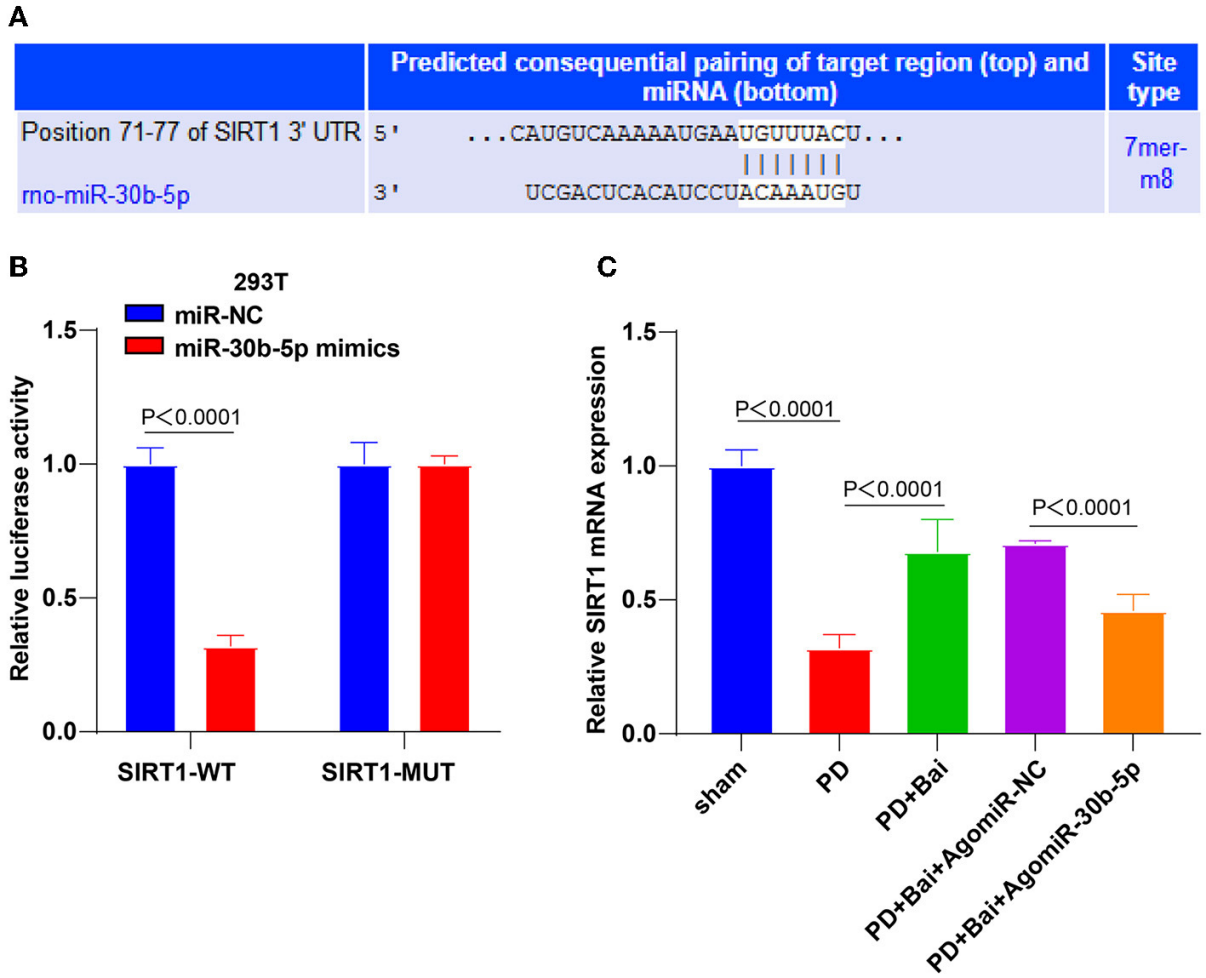
miR-30b-5p targeted SIRT1. **(A)** The binding sites between miR-30b-5p and SIRT1 were predicted by TargetScan website; **(B)** the target binding relationship between miR-30b-5p and SIRT1 was verified by dual-luciferase assay; **(C)** the expression of SIRT1 was detected by RT-qPCR. Three independent repeated cell tests were performed. *N* = 6. The data in the figure are all measurement data, expressed as mean ± standard deviation, and analyzed by one-way ANOVA and Tukey's multiple comparisons test.

To study how miR-30b-5p participated in the regulation of autophagy, the targeted binding sites between miR-30b-5p and SIRT1 were predicted through the bioinformatics online website Transtarget 7.2 (Figure 3A). The regulation of SIRT1 in autophagy is repeatedly reported (23, 24, 35). The binding relationship between miR-30b-5p and SIRT1 was elucidated by a dual-luciferase assay (Figure 3B). Finally, the relative expression of SIRT1 in rats was assessed by RT-qPCR, which showed repressed SIRT1 mRNA level in the PD group, whereas baicalein elevated SIRT1 level, and miR-30b-5p overexpression repressed SIRT1 (Figure 3C). These results manifested that miR-30b-5p targeted SIRT1.

### Knockdown of SIRT1 Partially Averted Baicalein-Induced Mitochondrial Autophagy

**Figure 4:**
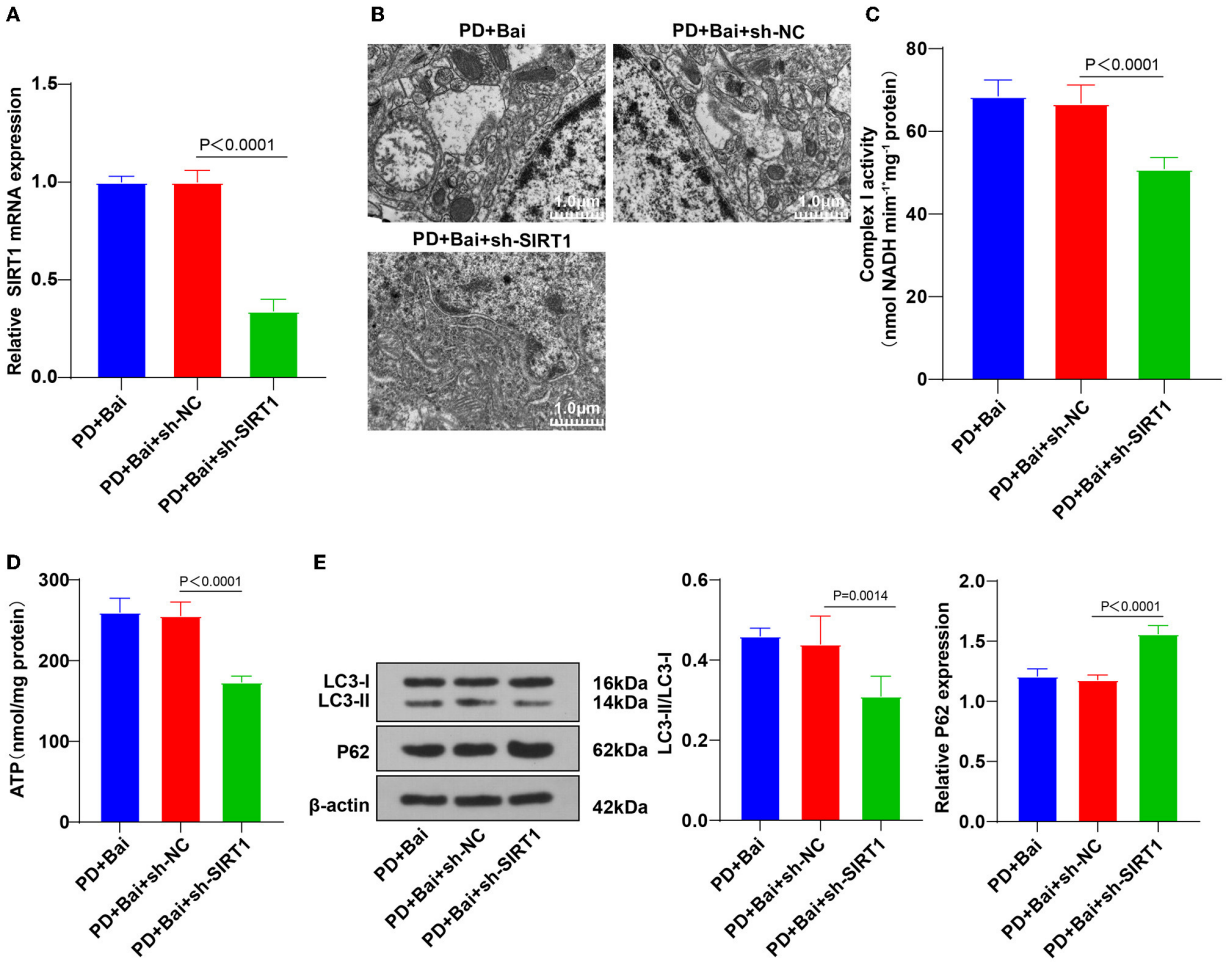
Downregulation of SIRT1 reversed baicalein-induced mitochondrial autophagy. The effect of SIRT1 on baicalein-induced mitochondrial autophagy was observed by inhibiting the expression of SIRT1 in PD rats by sh-SIRT1. **(A)** The expression of SIRT1 was detected by RT-qPCR; **(B)** the ultrastructure of mitochondria was observed using a TEM; **(C,D)** the activities of mitochondrial complex I and ATP were detected using kits; **(E)** the levels of autophagy marker proteins LC3-II/I and p62 were detected by WB. *N* = 6. The data in the figure are all measurement data and expressed as mean ± standard deviation; one-way ANOVA was used for variance analysis among multigroups; Tukey's multiple comparisons test was used for the *post hoc* test.

To further identify the mechanism of SIRT1 on baicalein-mediated mitochondrial autophagy, SIRT1 expression was silenced in PD rat brains by intracerebroventricular injection of sh-SIRT1 (Figure 4A), followed by baicalein treatment. Subsequently, the ultrastructure of mitochondria was observed using a TEM, the activities of mitochondrial complex I and ATP were detected, and the levels of LC3 and p62 were detected by WB. SIRT1 silencing partially annulled the property of baicalein on improving mitochondrial function (Figures 4B–D), raising LC3-II/LC3-I, and diminishing p62 (Figure 4E). Collectively, the knockdown of SIRT1 annulled the properties of baicalein in improving mitochondrial disorder and activating mitochondrial autophagy.

### Baicalein Stimulated Mitochondrial Autophagy *via* miR-30b-5p and the SIRT1/AMPK/MTOR Pathway

**Figure 5:**
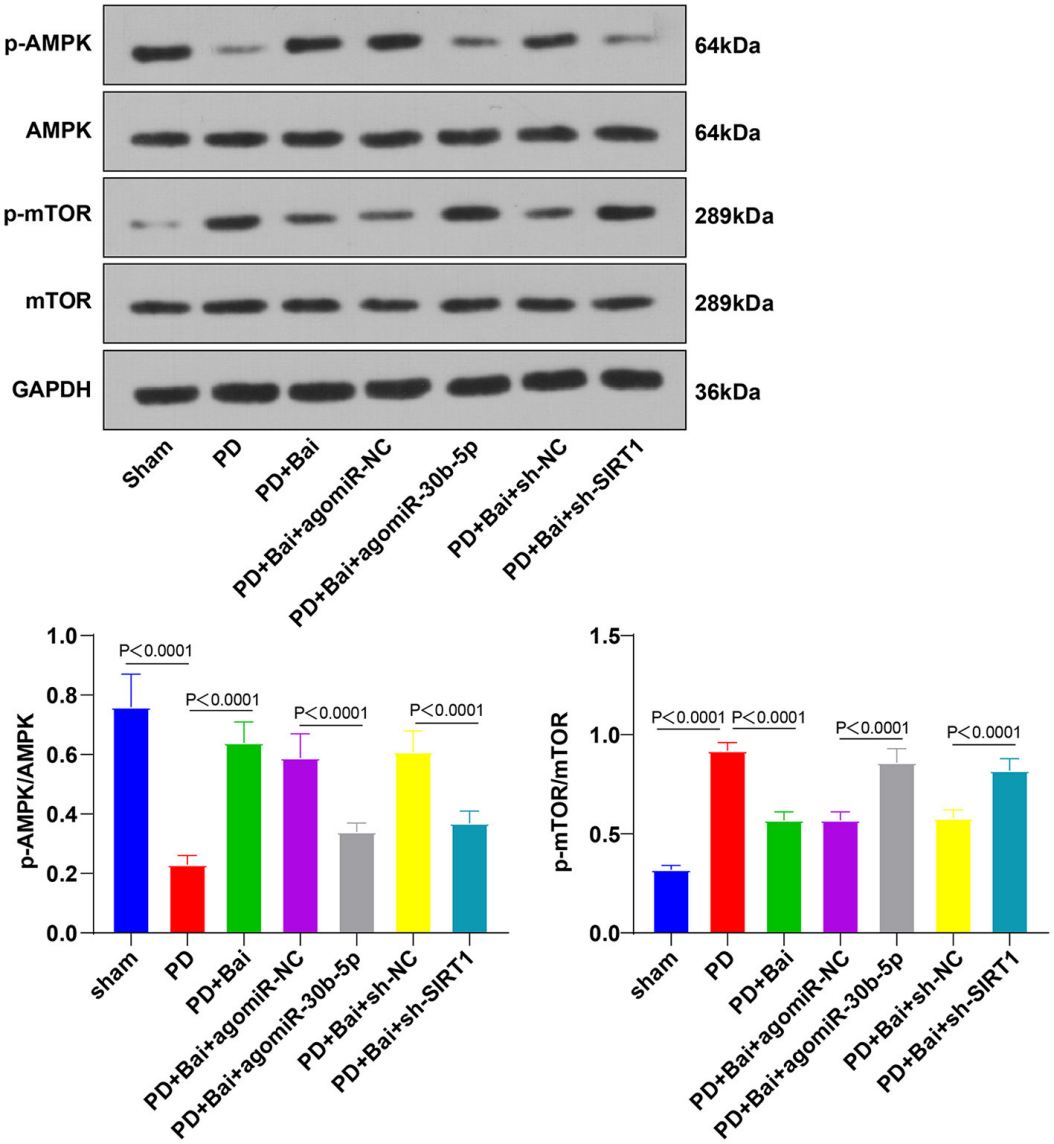
Baicalein mediated mitochondrial autophagy *via* miR-30b-5p/SIRT1 and the AMPK/mTOR signaling pathway. The effect of miR-30b-5p on the AMPK/mTOR signaling pathway was observed by transfecting agomir-30b-5p in PD rats. The phosphorylation levels of AMPK and mTOR were detected by WB. *N* = 6. The data in the figure are all measurement data and expressed as mean ± standard deviation; one-way ANOVA was used for variance analysis among multigroups; Tukey's test was used for the *post hoc* test.

The AMPK/mTOR pathway is a pivotal pathway to manipulate mitochondrial autophagy. SIRT1 activates the AMPK/mTOR pathway (25, 36–38). Thereby, we postulated that baicalein manipulated mitochondrial autophagy *via* the AMPK/mTOR pathway and the miR-30b-5p/SIRT axis. To verify this, the activation of AMPK and mTOR was assessed by WB. As a result, the phosphorylation level of AMPK was suppressed, and mTOR was stimulated in the PD group, whereas baicalein raised the phosphorylation level of AMPK and decreased mTOR. Overexpression of miR-30b-5p or knockdown of SIRT1 partially annulled the role of baicalein in the AMPK/mTOR pathway (Figure 5). Altogether, baicalein stimulated mitochondrial autophagy *via* the miR-30b-5p/SIRT1/AMPK/mTOR axis.

## Discussion

Parkinson's disease is the most severe and prevalent neurodegenerative disorder worldwide, and the prevalence will surge as the population ages (39). A prior study has elicited the neuroprotection of baicalein on PD (40). This study evaluated the functional mechanism of baicalein in PD and elucidated that baicalein was neuroprotective in PD by stimulating mitochondrial autophagy *via* miR-30b-5p and the SIRT1/AMPK/mTOR pathway.

In PD patients, rehabilitation of neurological function can effectively prevent further deterioration of the condition (41). The dopaminergic neuron loss is responsible for PD motor symptoms (42). Baicalein protects nerve cells against E46K alpha-synuclein toxicity in PD cell models (31). Limited DA contents closely link with PD development (43). Our results demonstrated that PD rats elicited severe neurological decline, decreased related-motor and sensory coordination and DA content in the striatum, increased neuron apoptosis, and aggravated neuronal injury, whereas after baicalein treatment, these trends were partially alleviated. Consistently, a high dose of baicalein significantly alleviated the reduction in DA content in the striatum (28). Baicalein possesses the pharmacological function of neurogenesis and antiapoptotic effect (16). A previous study has shown that baicalein (concentration 30 mg/kg) can cross the blood–brain barrier after intravenous injection (44). Fong SY et al. have detected the content of 6 kinds of baicalein metabolites after oral administration using the SPE-LC/MS/MS method and found that their contents in rat brain tissue were increased, which strongly confirm that baicalein can cross the blood-brain barrier (45). A recent study has shown that baicalein has the best physical and chemical properties in oral bioavailability and blood-brain barrier permeability (46). These studies have shown that baicalein can cross the blood–brain barrier to exert its function. In brief, baicalein had a protective function in PD rats.

Mitochondrial autophagy is a critical process in PD (9). Our results showed swelling, unclear double-layer membrane structure, and blurred and disordered arrangement of mitochondrial ridge in mitochondria of neurons in substantia nigra of PD rat brain, whereas baicalein treatment restored mitochondria number and the morphology in PD rats. The deficiency of complex I and ATP can manifest as a mitochondrial disease (47). Our results elicited that complex I and ATP activities were repressed in PD rats, whereas baicalein treatment partially restored the activities. Consistently, baicalein pretreatment increased the levels of ATP and complex I (48). In conclusion, baicalein improved mitochondrial dysfunction of neurons in substantia nigra of PD rats. The alteration of mitochondrial autophagy is involved in neurodegenerative diseases, especially PD (33). LC3 and P62 proteins are detected to reflect the autophagic flux (49), and LC3-II/LC3-I is suggestive of autophagy status (50). Our results elicited decreased LC3-II/I ratio and increased p62 level in neurons in substantia nigra of PD rats, indicating decreased mitochondrial autophagy, whereas baicalein partially restored mitochondrial autophagy. Consistently, LC3-II/LC3-I is facilitated (51) and p62 expression is suppressed in PD (52). Baicalein reduces LC3-II/LC3-I in ischemic stroke (53) and elevates p62 level in acute liver injury (54). Baicalein is also reported to prevent neurotoxicity by restoring autophagy (55). To ascertain the role of autophagy in baicalein-mediated protection, we treated PD rats with autophagy inhibitor 3-MA and baicalein and noted that the protection of baicalein in PD rats was suppressed. In consistency, 3-MA can inhibit baicalein-induced autophagy, thus worsening liver injury (56). In summary, the protection mechanism of baicalein on PD rats might link with mitochondrial autophagy.

The miR-30 family is abnormally expressed in PD (22). hsa-miR-30b-5p has principal effects on neurodegenerative diseases and is reported as a biomarker for PD (21). Our results demonstrated upregulated miR-30b-5p in the PD rats, whereas baicalein treatment inhibited its expression. Consistently, clear overexpression of miR-30b-5p is observed in PD patients (22). Then, we overexpressed miR-30b-5p in PD rats and treated them with baicalein. Expectedly, miR-30b-5p overexpression nullified the property of baicalein on improving mitochondrial function and inhibiting neuronal autophagy in PD rats. However, no study, to our knowledge, has considered the function of baicalein in miR-30b-5p regulation. This study initially highlighted that baicalein repressed miR-30b-5p and miR-30b-5p overexpression inhibited baicalein-stimulated mitochondrial autophagy.

To validate the mechanism of miR-30b-5p on mitochondrial autophagy, we predicted the downstream targets of miR-30b-5p and obtained SIRT1. The binding relationship was verified. Our results showed that SIRT mRNA level was suppressed in PD rats, elevated by baicalein treatment, and diminished by miR-30b-5p overexpression. Consistently, the enzymatic activity of SIRT1 is reduced in PD patients, which reduces the ability to resist neurotoxin-induced neuronal injury (57). Collectively, miR-30b-5p targeted SIRT1. To identify the role of SIRT1 in baicalein-mediated mitochondrial autophagy, we silenced SIRT1 in PD rats and treated PD rats with baicalein and discovered that SIRT1 knockdown partially averted the function of baicalein on improving mitochondrial function, elevating LC3-II/LC3-I, and lowering p62. Consistently, knockdown of SIRT1 averted melatonin-mediated attenuation of the NLRP3 inflammasome activation and aggravated PD (58). In brief, SIRT1 silencing averted the property of baicalein on promoting mitochondrial autophagy and improving mitochondrial disorder.

The AMPK/mTOR pathway is implicated in autophagy and neuronal apoptosis regulation in PD (59). Thereby, we guessed that baicalein modulated mitochondrial autophagy through the AMPK/mTOR pathway. Subsequent results elicited that the phosphorylation level of AMPK was repressed and mTOR was facilitated in PD rats, whereas baicalein elevated the phosphorylation level of AMPK and suppressed mTOR. miR-30b-5p overexpression and SIRT1 silencing partially annulled the function of baicalein on regulating the AMPK/mTOR pathway. circSAMD4A plays a role in the autophagy and apoptosis of the dopaminergic neurons *via* the AMPK/mTOR pathway in PD (60). Collectively, baicalein manipulated mitochondrial autophagy *via* the miR-30b-5p/SIRT1/AMPK/mTOR axis.

To conclude, this study elicited that baicalein protected PD rats by stimulating mitochondrial autophagy *via* miR-30b-5p and the SIRT1/AMPK/mTOR pathway. However, whether baicalein mediates other mitochondrial autophagy pathways and whether these findings can provide baicalein-targeted approaches for the clinical treatment of PD have not been studied. Further investigation is warranted to deeply search the clinical approaches for PD from the aspect of baicalein.

## Data Availability Statement

The original contributions presented in the study are included in the article/Supplementary Material, further inquiries can be directed to the corresponding author.

## Ethics Statement

The animal study was reviewed and approved by the academic Ethics Committee of Taihe Hospital.

## Author Contributions

MC and LP performed conceptualization. PG, XZ, and TS involved in validation, research, resources, data reviewing, and writing. XZ and JH done reviewing and editing. All authors read and approved the final manuscript.

## Funding

This research was supported by funds from the Hubei Provincial Education Department Scientific Research Guidance Project in 2015 (B2015473).

## Conflict of Interest

The authors declare that the research was conducted in the absence of any commercial or financial relationships that could be construed as a potential conflict of interest.

## Publisher's Note

All claims expressed in this article are solely those of the authors and do not necessarily represent those of their affiliated organizations, or those of the publisher, the editors and the reviewers. Any product that may be evaluated in this article, or claim that may be made by its manufacturer, is not guaranteed or endorsed by the publisher.

## Supplementary Material

Figure S1 Baicalein played a protective role in the PD rat model. PD model operation was performed in SD rats, and the protective effect of baicalein on PD rats was observed after baicalein treatment. **(A)** Neurological score; **(B)** DA content was detected by HPLC-EC; **(C)** apoptosis was detected by TUNEL staining; **(D)** neuronal degeneration was detected by Fluoro-Jade B staining. *N* = 6. The data in the figure are all measurement data and expressed as mean ± standard deviation; one-way ANOVA was used for variance analysis; Tukey's multiple comparisons test was used for the *post hoc* test. ^***^*p* < 0.001.
