# Supplementary figures and images for: Baicalein Induces Mitochondrial Autophagy to Prevent Parkinson's Disease in Rats via miR-30b and the SIRT1/AMPK/mTOR Pathway
Source: Front Neurol. 2022 Feb 14;12:646817. doi: 10.3389/fneur.2021.646817 (PMC8883053; doi:10.3389/fneur.2021.646817)

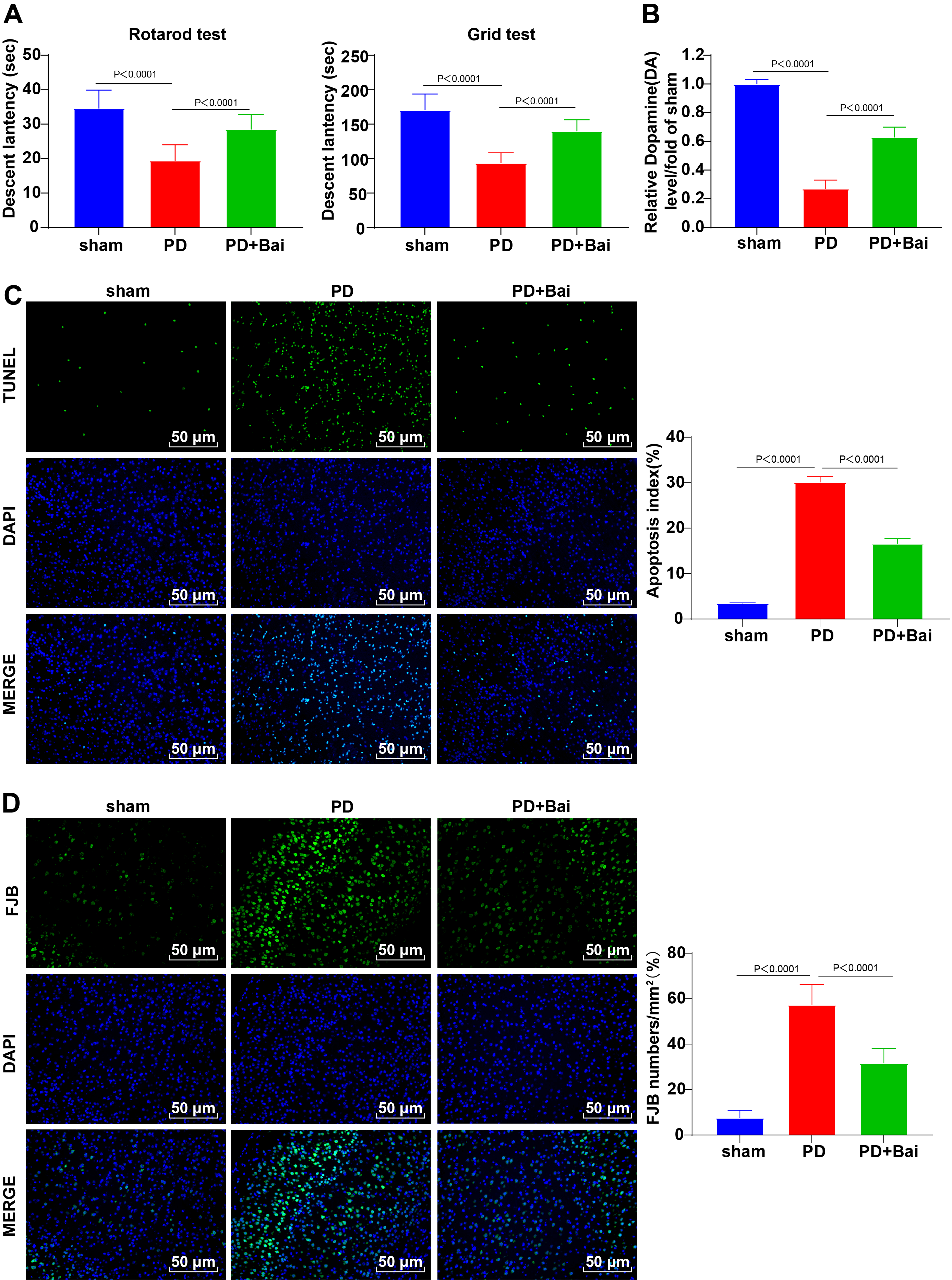

Supplement: Figure S1 — Baicalein played a protective role in the PD rat model. PD model operation was performed in SD rats, and the protective effect of baicalein on PD rats was observed after baicalein treatment. (A) Neurological score; (B) DA content was detected by HPLC-EC; (C) apoptosis was detected by TUNEL staining; (D) neuronal degeneration was detected by Fluoro-Jade B staining. N = 6. The data in the figure are all measurement data and expressed as mean ± standard deviation; one-way ANOVA was used for variance analysis; Tukey's multiple comparisons test was used for the post hoc test. ***p < 0.001. [file Image_1.TIFF]
